# Case report: An unusual presentation of intra-abdominal desmoplastic small round cell tumor

**DOI:** 10.3389/fonc.2024.1260474

**Published:** 2024-02-19

**Authors:** Akshay Nilesh Desai, Christine Jane Kurian, William Rafferty, Danielle Lajoie Behrens, Polina Khrizman

**Affiliations:** ^1^ Department of Internal Medicine at Cooper University Healthcare, Camden, NJ, United States; ^2^ Department of Medical Oncology and Hematology at MD Anderson Cancer Center at Cooper University Healthcare, Camden, NJ, United States; ^3^ Department of Pathology at Cooper University Healthcare, Camden, NJ, United States

**Keywords:** desmoplastic small round cell tumor, sarcoma, case report, EWSR-WT1, pathology, next generation sequencing

## Abstract

**Background:**

Intra-abdominal desmoplastic small round cell tumor (IDSRCT) is a rare entity (0.2–0.74 cases per million people per year), which predominantly occurs in young men. It may present as an abdominal mass with pain, distention, and constipation. IDSRCT has a very poor prognosis, with 5-year overall survival estimated at 15%–30%. Diagnosis is made with tissue biopsy.

**Case description:**

We present a case of a 28-year-old man with a history of schizophrenia and depression who presented to an emergency room (ER) in November 2022 with constipation and pelvic pain. The patient was sent home with a bowel regimen after radiography showed no obstruction. He re-presented for evaluation due to persistent pain. A computerized tomography scan of the abdomen and pelvis (CT A/P) revealed numerous pelvic masses with severe colitis, bilateral moderate hydronephrosis, and metastatic disease in the liver. A colonoscopy showed a mass extending 3 cm from the anus to 10 cm causing a partial obstruction. Biopsy was read as squamous cell carcinoma (SCC). The patient was subsequently admitted to our institution with pelvic pain, nausea, and vomiting. Colorectal surgery performed a colectomy with end-ileostomy due to colonic obstruction. He was evaluated by a medical oncologist, with previous slides requested for review. Initial review was concerning metastatic basaloid SCC with neuroendocrine features and a Ki67 of 70%. Given his recent abdominal surgeries, chemotherapy was delayed until February 2023 when he was started on reduced dose carboplatin and paclitaxel. Tumor specimen was sent for next generation sequencing (NGS) and programmed death-1 ligand 1 (PD-L1) testing. NGS results returned after the first dose of chemotherapy was given and showed a t(11;22) EWSR-WT1 translocation characteristic of desmoplastic small round cell tumor. The patient was supported in the hospital and discharged with oncology follow-up.

**Discussion:**

As seen in this case, pathology review is essential to ensuring correct diagnosis and appropriate treatment plan. This is especially true when the clinical scenario does not match the listed pathology. Additional diagnostics such as NGS are invaluable in establishing correct diagnosis.

## Introduction

Desmoplastic small round cell tumor (DSRCT) is a rare subtype of sarcoma that often develops in the abdomen. It was first characterized in 1989 by Gerald and Rosai (1), who discovered the t (11;22) (p13;q12) translocation resulting in the formation of EWSR1-WT1 gene fusion. EWSR1-WT1 gene fusion is pathognomonic for this disease, as the translocation upregulates expression of platelet-derived growth factor receptor A (PDGFRα), vascular endothelial growth factor (VEGF), and other proteins (2–4). DSRCT has a wide age range from 5 years to 50 years, with 85%–90% of cases occurring in men. Prognosis for the disease is very poor, with 5-year overall survival estimated at 15%–30% (4). Here, we present a case of a young man who developed sudden, nonspecific abdominal pain and was eventually diagnosed with intra-abdominal desmoplastic small round cell tumor.

## Case description

**Figure 1:**
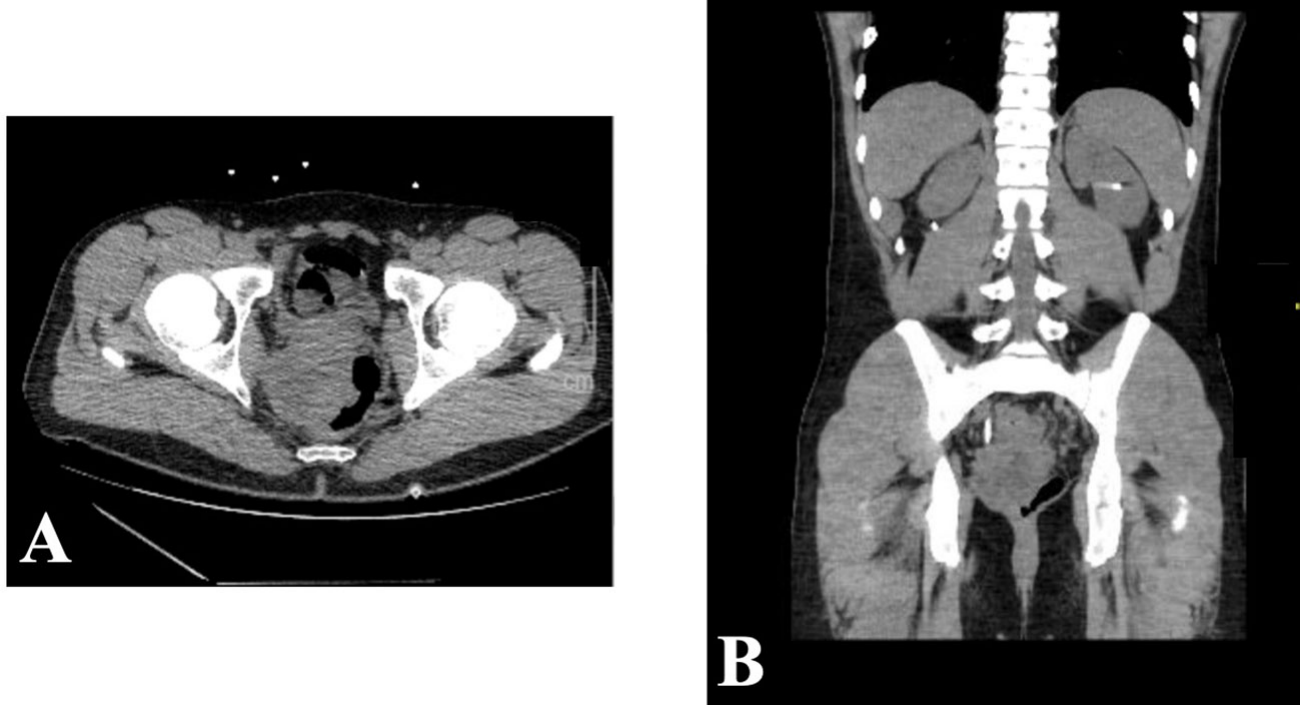
November 2022 CT abdomen and pelvis revealing rectal mass in both axial **(A)** and sagittal **(B)** views.

A 28-year-old man with a past medical history of schizophrenia and depression initially presented in November 2022 to an outside institution’s emergency department with constipation and abdominal pain. He underwent abdominal X-ray, which was negative for obstruction and was sent home with a bowel regimen. Despite this, his pain persisted, and he presented for repeat evaluation. He underwent CT A/P, which revealed numerous pelvic masses with severe colitis, bilateral moderate hydronephrosis, and metastatic disease in the liver (**Figure 1**). Carcinoembryonic antigen (CEA) was 2.4 ng/mL, alpha fetoprotein (AFP) was 2.5 ng/mL, and prostate-specific antigen (PSA) was 0.6 ng/mL. He underwent bilateral percutaneous nephrostomy tube placement to improve his hydronephrosis. Shortly afterwards, the patient underwent a colonoscopy that revealed a mass extending 3 cm from the anus to 10 cm, causing a partial obstruction. Biopsy was obtained with initial pathology read as squamous cell carcinoma (SCC).

The patient met with an oncologist who reviewed the case at tumor board, with a decision to pursue palliative chemotherapy with carboplatin and paclitaxel for presumed metastatic SCC of rectal origin. Repeat staging CT chest, abdomen, and pelvis (CT C/A/P) showed a large rectal mass with peritoneal carcinomatosis and liver metastatic disease. This also noted extensive lymphadenopathy and a sclerotic bone lesion in the right iliac.

## Diagnostic assessment

In January 2023, the patient was admitted at our institution with pelvic pain, nausea, vomiting, and suicidal ideation. Colorectal surgery was consulted, and a colectomy with end-ileostomy was performed due to colonic obstruction secondary to the mass. His hospital course was subsequently complicated by abdominal wound dehiscence and evisceration, and he was brought back to the operating room (OR) for closure. During this admission, medical oncology was consulted, and previous slides were requested. Initial review by our institution’s pathology department was concerning basaloid SCC with neuroendocrine features, and a Ki67 of 70%. Given his recent abdominal surgeries, chemotherapy was delayed until 14/02/2023 when he was started on reduced dose carboplatin and paclitaxel. He received supportive growth factor on 16/02/23. Additionally, he was started on total parenteral nutrition (TPN) due to poor appetite following his multiple abdominal surgeries and peritoneal carcinomatosis.

**Figure 2:**
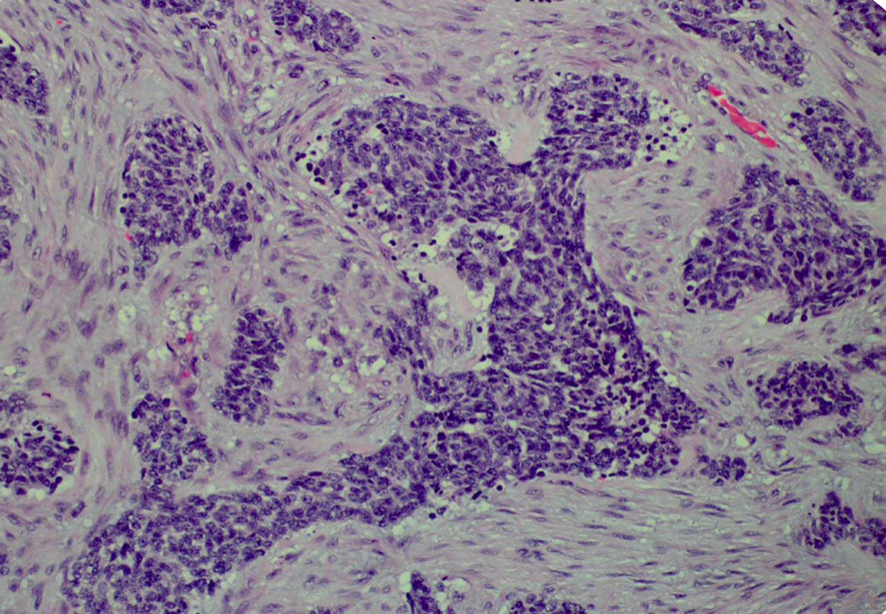
Nests of small round blue cells separated by dense fibrous stroma (×200).

**Figure 3:**
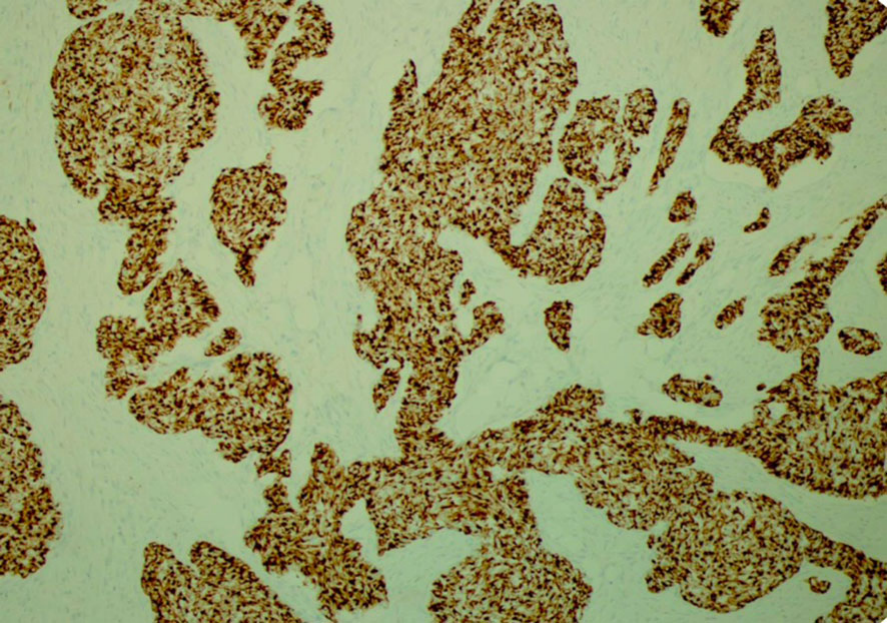
Strong desmin reactivity characteristic of desmoplastic small round cell tumor (×200).

**Figure 4:**
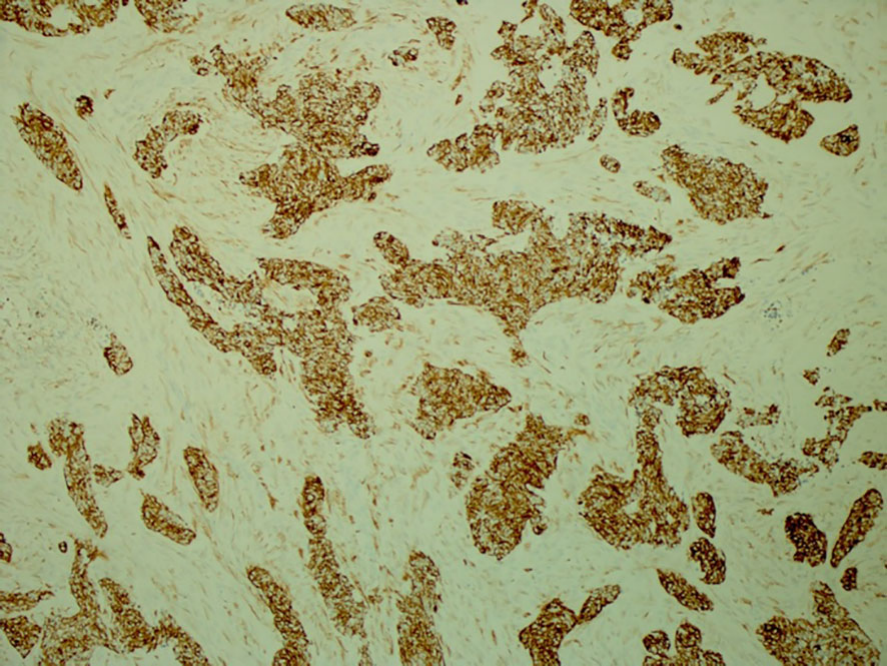
Positive keratin staining (CAM 5.2) highlighting the polyphenotypic immunoprofile of this tumor with expression of epithelial and mesenchymal markers (×100).

His tumor specimen was sent for NGS and PD-L1 testing. NGS results returned after first dose of chemotherapy was given, which showed a t(11;22) EWSR-WT1 translocation, which is a characteristic of desmoplastic small round cell tumor. Pathology was closely reviewed, and it revealed nests of round blue cells separated by desmoplastic stroma (**Figure 2**), cells with strong desmin reactivity (**Figure 3**), and positive keratin staining (CAM, 5.2) (**Figure 4**). These findings were highly characteristic of DSRCT. No further carboplatin and paclitaxel were given. He was closely monitored during his hospitalization and eventually discharged with plan for close oncological follow-up for intra-abdominal DSRCT. Currently, the patient is on palliative vincristine (2 mg), doxorubicin (37.5 mg/m^2^), and cyclophosphosphamide (1200 mg/m^2^) (VAC) every 3 weeks. He has completed a total of five cycles of treatment.

## Discussion

DSRCT is a very rare subtype of sarcoma, with just 0.2–0.74 cases per million per year. In fact, a 2014 study identified just 192 cases of DSRCT worldwide in the Survival, Epidemiology, and End Results (SEER) database (5). As previously noted, the hallmark characteristic of DSRCT is the EWSR1-WT1 gene fusion. The chimeric product of the EWS-WT1 fusion protein acts as a major transcriptional activator factor that results in the upregulation of PDGFRα, VEGF, and other proteins related to tumor and vascular cell progression (6, 7). Upon recognition of DSRCT, management entails a combination of chemotherapy, radiation, and aggressive cytoreductive surgery (4). However, despite several advances in the multimodal therapy approach employed to treat DSRCT, outcomes are unfortunately poor, as the expected survival time after diagnosis approaches 3 years. Because of DSRCT’s rarity and its overall poor prognosis, the recognition of the disease is paramount to ensure appropriate treatment.

Our patient underwent biopsy of a colonic mass that was initially determined to be squamous cell carcinoma, negative for cytokeratin 7 and cytokeratin 20 (CK7, CK20). Palliative carboplatin and paclitaxel were planned for the treatment of the patient’s presumed rectal squamous cell carcinoma, with CT imaging confirming large rectal mass with peritoneal carcinomatous. This scan also revealed extensive lymphadenopathy and a sclerotic bone lesion in the right iliac. However, treatment was never started, as the patient and his family desired for a second opinion.

Our patient’s initial presentation and subsequent treatment plan highlight the need for additional diagnostic testing in the setting of unclear pathology. Typically, DSRCT originates from the peritoneum or retroperitoneum and can subsequently invade the omentum. Numerous peritoneal implants can subsequently be discovered in the diaphragm, pelvic peritoneum, and mesentery of the small and large bowel (8, 9). Other sites of primary tumor have been noted in a variety of locations, including the thoracic cavity, thigh, and skull (10). Most commonly, imaging will note multiple lobulated, heterogeneous peritoneal, omental, and serosal soft tissue masses without an apparent primary organ of origin (11, 12). While our patient did present with some classic symptoms including abdominal pain, distension, and constipation, his initial scan noted a large lobulated mass 6 × 7 cm, located in the rectosigmoid junction. Thus, his presentation and location was initially most concerning for metastatic disease of rectal primary as opposed to a diffusely metastatic sarcoma.

Histologically, DSRCT is noted to have solid sheets or large nests of small round cells, noted to have inconspicuous nucleoli (13). Immunohistochemistry can reveal expression of desmin and neural markers such as neuron-specific enolase and CD57. Furthermore, the differential diagnosis of DSRCT includes a spectrum of other round cell neoplasms, including rhabdomyosarcoma, small cell carcinoma, and Ewing sarcoma. As the diagnosis of DSRCT is made via a combination of the histological appearance and immunohistochemical staining, hallmark histological features in core biopsy specimens may not be appreciated easily. In our patient’s case, initial histology was concerning squamous cell that, upon further review at our institution, had evidence of neuroendocrine features (14). Our patient’s true diagnosis of DSRCT was not revealed until NGS returned with the pathognomonic t(11;22) EWSR-WT1 translocation. Given the updated pathological findings, Ewing sarcoma treatment with VAC was initiated. This underscores that pathology review and re-examination is crucial to ensuring correct diagnosis and appropriate treatment plan.

## Conclusion

Desmoplastic small round cell tumor (DSRCT) is an exceedingly rare sarcoma subtype most commonly seen in young men. It has an extremely poor prognosis, thus making accurate diagnosis paramount. While it typically presents with several peritoneal implants and usually does not have any radiographic evidence suggestive of a primary organ of origin, our patient demonstrates that cases of DSRCT can present atypically, which can make precise diagnosis difficult. As such, clinicians should have a keen index of suspicion in patients when a patient’s clinical scenario does not match the patient’s listed pathology.

## Patient perspective

Written informed consent was obtained from the patient on 14/07/2023.

## Data availability statement

The original contributions presented in the study are included in the article/**Supplementary Material**. Further inquiries can be directed to the corresponding author.

## Ethics statement

Written informed consent was obtained from the individual(s) for the publication of any potentially identifiable images or data included in this article.

## Author contributions

AD: Writing – original draft, Writing – review & editing, Conceptualization, Investigation. CK: Conceptualization, Writing – review & editing, Writing – original draft, Supervision, Validation. WR: Formal analysis, Investigation, Methodology, Supervision, Writing – review & editing, Validation. DB: Conceptualization, Investigation, Supervision, Writing – review & editing, Validation. PK: Investigation, Supervision, Writing – review & editing, Validation.
