# Supplementary figures and images for: Case report: An unusual presentation of intra-abdominal desmoplastic small round cell tumor
Source: Front Oncol. 2024 Feb 19;14:1260474. doi: 10.3389/fonc.2024.1260474 (PMC10910504; doi:10.3389/fonc.2024.1260474)

## Supplemental Figure 1: Relevant timeline of events

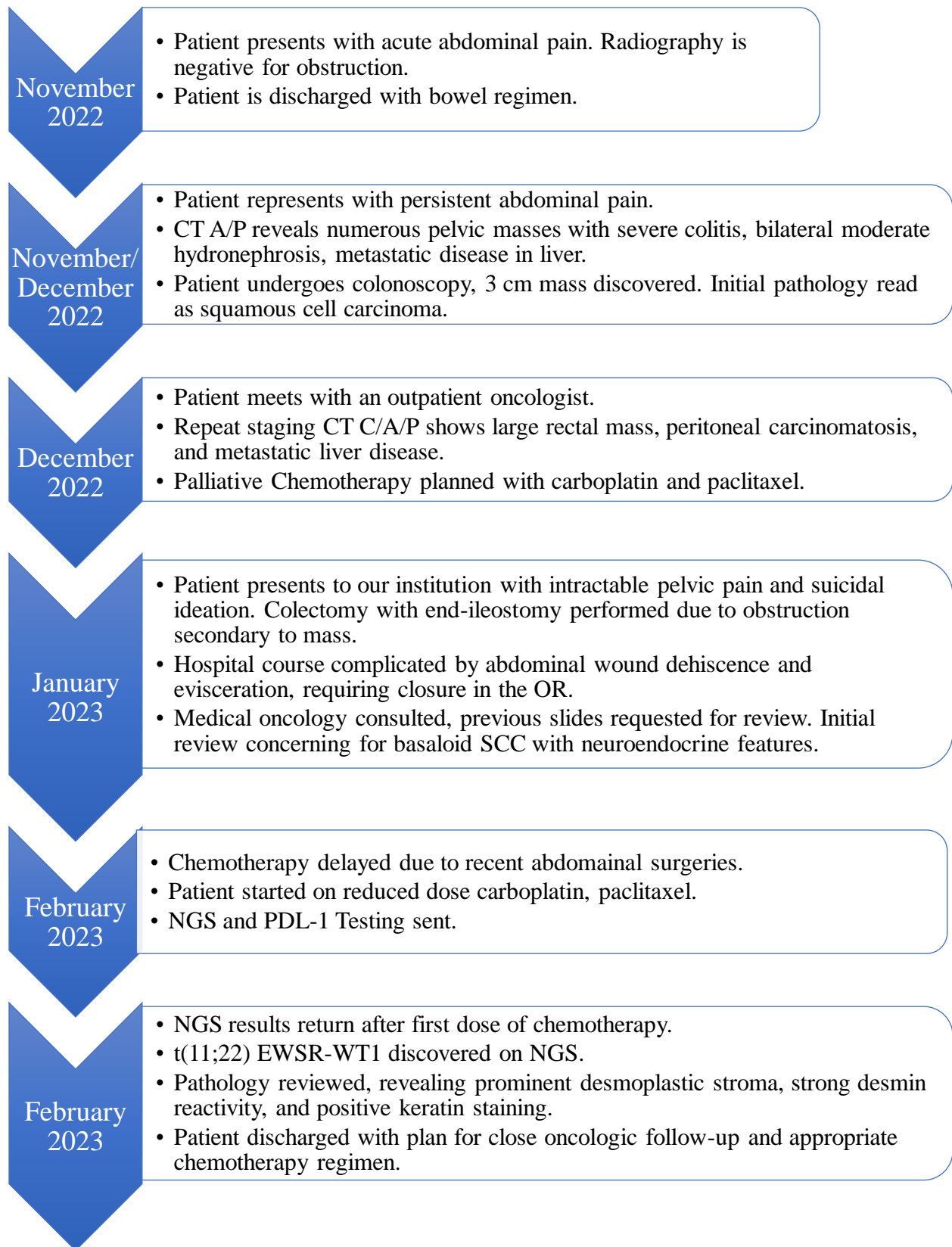

Supplement: Supplementary file 2 [file Image_1.pdf]
